# Circularly Polarized MIMO Antenna with Wideband and High Isolation Characteristics for C-Band Communication Systems

**DOI:** 10.3390/mi13111894

**Published:** 2022-11-02

**Authors:** Niamat Hussain, Tuyen Danh Pham, Huy-Hung Tran

**Affiliations:** 1 Department of Smart Device Engineering, Sejong University, Seoul 05006, Korea; 2 IT Department, FPT University, Hanoi 11312, Vietnam; 3 Faculty of Electrical and Electronic Engineering, PHENIKAA University, Hanoi 12116, Vietnam; 4 PHENIKAA Research and Technology Institute (PRATI), Hanoi 11313, Vietnam

**Keywords:** MIMO antenna, circularly polarized, wideband, high isolation, patch, parasitic element

## Abstract

This paper presents a circularly polarized (CP) multiple-input multiple-output (MIMO) antenna using a microstrip patch and parasitic elements. The proposed design exhibits wideband characteristics for both impedance and axial ratio bandwidths. Especially, the mutual coupling between the MIMO elements is significantly depressed without using any decoupling network. To achieve these features, parasitic elements are positioned nearby and in different layers to the radiating elements. The measured results demonstrate that the proposed MIMO CP antenna has a wideband operation of 11.3% (5.0–5.6 GHz), which is defined by an overlap between −10–dB impedance and 3–dB axial ratio bandwidths. Across this band, the realized gain is better than 6.0 dBi, and the isolation is greater than 32 dB with the highest value of 45 dB. The MIMO parameters such as the envelope correlation coefficient, diversity gain, and channel capacity loss are also investigated thoroughly, which are found to be good on the scale of diversity standards.

## 1. Introduction

Multiple-input multiple-output (MIMO) antennas have been widely used in modern wireless communication systems due to their increased link capacity [1]. The multiple antennas at the receiver and transmitter provide several paths for signal transmission to avoid seamless connectivity. In comparison with linearly polarized (LP) antennas, circularly polarized (CP) antennas are more beneficial and they have been demonstrated as a potential candidate for MIMO systems [2]. The CP MIMO antennas are robust against transmission losses and offer additional freedom for the antenna’s orientation which are critical requirements in the 5G IoTs and randomly oriented radio frequency and identification tags. The improvement of the overlapping bandwidth (BW), defined by 3-dB axial ratio (AR) and −10 dB impedance BWs, with improved isolation has always been a challenge. Several topologies have been adopted to increase overlapping BW and isolation in MIMO antennas. However, it always comes with a tradeoff, either with antenna profile, complex geometry, or low mechanical rigidity. This paper focuses on designing a MIMO CP antenna with the wideband operation, unidirectional radiation pattern, as well as planar configuration. The reason for the wideband feature is that modern wireless devices always require fast communication. Meanwhile, the planar geometry can be integrated conveniently into the devices.

In [3,4,5], the authors have proposed a method to achieve wideband performance using a dielectric resonator antenna (DRA). Alternatively, a combination of the patch antenna and frequency selective surface (FSS) is another effective solution [6,7]. However, these designs often require a very high profile and/or very large lateral dimensions, which cause lots of difficulties for device integration.

The microstrip patch antenna is the best solution to achieve simultaneously wideband and planar configuration. Several MIMO antennas employing multiple CP radiating patches have been reported in [8,9,10,11]. Nonetheless, their narrow operating BW makes them less attractive to wideband systems. To broaden BW, a technique of using metasurface (MS) is proposed in [12,13]. Alternatively, wideband can also be attained when combining patch and parasitic elements [14].

Apart from operating BW, the isolation among the MIMO elements is another critical task in designing the MIMO antenna. The techniques to reduce the mutual coupling can be classified into three types: defected ground structure (DGS) [15,16,17], complicated decoupling network [18,19,20], and near–field resonant structure [21,22,23]. It is worth noting that in the most common of these methods, the additional structures only have one function of mitigating mutual coupling.

In this paper, a MIMO CP patch antenna with wideband and high isolation characteristics is proposed. Unlike the methods employed in [15,16,17,18,19,20,21,22,23], an additional structure comprising multiple parasitic elements is employed to enhance not only the isolation but also the operating BW. For a better demonstration of the advantages of the presented work, Table 1 shows a performance comparison among the MIMO CP antenna in the literature. λ_o_ is the free–space wavelength at the lowest operating frequency. As observed, the proposed design possesses high isolation over a wide frequency band while keeping small element spacing and low-profile configuration. Although the designs in [12,13] can achieve wider BW and higher gain, it requires much larger element spacing. Besides, it is worth noting that the proposed design has the best isolation over the operating BW.

## 2. Antenna Geometry

**Figure 1:**
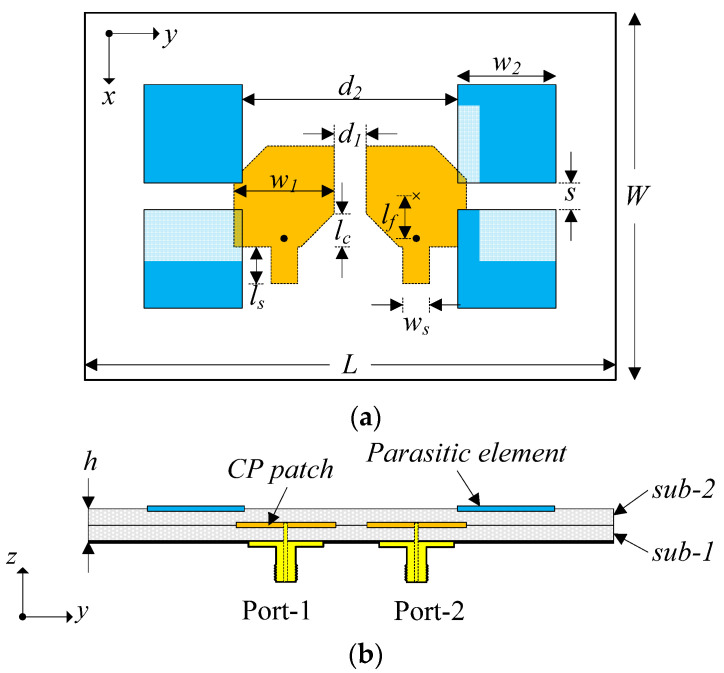
Geometry of the proposed MIMO CP antenna: (**a**) Top–view; (**b**) Cross–section view.

Figure 1 shows the geometry of the proposed MIMO CP antenna in the top view and cross-section view. The antenna is printed on two 1.52–mm–thick Taconic TLY substrates with dielectric permittivity of *ε_r_* = 2.2. The CP sources are two microstrip square patches and the patch’s size (*w*_1_) is about half–effective wavelength at the desired frequency. By truncating different corners of the patches, dual–sense CP of right–hand CP (RHCP) and left–hand CP (LHCP) is generated. The radiating patches are printed at the top of a sub–1. For mutual coupling reduction and BW enhancements, four parasitic elements are positioned nearby the radiating patches and printed at the top of a sub–2. The size of the parasitic element is smaller than the size of the radiating element to broaden the operation in the high–frequency range. With respect to the impedance matching, the additional stub is inserted in each MIMO element. It is noted that these stubs only have a strong effect on the matching performance of the antenna. Finally, the antenna is fed by two 50–Ω coaxial cables, which go through the substrates and connect to the antenna.

## 3. Antenna Design Procedure

In order to provide a better understanding of the proposed MIMO CP antenna, the antenna design procedure is divided into three main steps to investigate. It is noted that the profile of the considered designs is kept identical at the optimized value (*h* = 3.04 mm) for a fair comparison. Besides, since the distance between the MIMO elements strongly affects the isolation of the system, the edge-to-edge spacing between two patches is fixed at the optimal value of d_1_ = 5 mm in all cases. The optimized dimensions for all designs are shown in Table 2.

### 3.1. MIMO Antenna without Parasitic Elements

**Figure 2:**
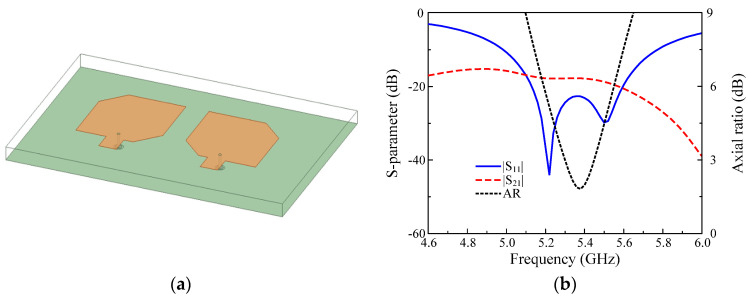
MIMO CP antenna without parasitic element: (**a**) 3D–view; (**b**) Simulated results.

Firstly, the antenna without parasitic elements, designated as Design–1, is considered. Figure 2a shows the configuration of Design–1. The antenna performances in terms of S–parameter and AR as well are also shown in Figure 2b. Generally, Design–1 has narrow band operation with −10 dB |S_11_| BW from 5.0 to 5.8 GHz and 3–dB AR BW from 5.3 to 5.45 GHz. In terms of isolation, the transmission coefficient |S_21_| is around –18 dB, which is quite high and might not be suitable for the MIMO system. In fact, this value can be improved when increasing the element spacing. However, this will increase the antenna’s lateral dimensions. It is noted that for MIMO antenna with LP elements, high isolation can be achieved when polarization diversity is met. However, this is completely different when deploying a MIMO antenna with CP elements. This has been thoroughly investigated and demonstrated in [14]. 

### 3.2. MIMO Antenna with Parasitic Elements and Radiating Patches in the Same Layer

**Figure 3:**
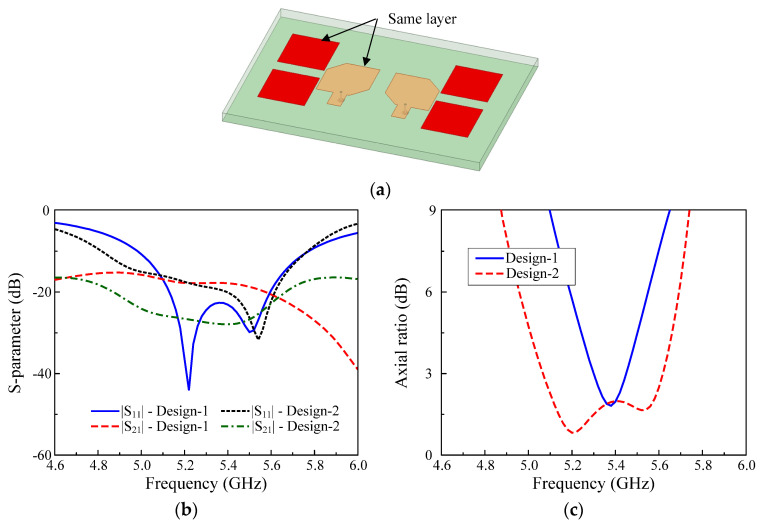
MIMO CP antenna with parasitic elements and radiating patches in the same layer: (**a**) 3D–view; (**b**) Simulated S–parameter; (**c**) Simulated axial ratio.

The MIMO antenna with parasitic elements as Design–2 is shown in Figure 3a. Here, four parasitic elements are employed, and they are arranged on only one side of the radiating patch. Note that in this case, the radiating patches and the parasitic elements are located in the same layer.

Figure 3b,c show the performance comparison between Design–1 and –2. It can be seen that the use of parasitic elements can significantly improve the operating BW from 2.8 to 10.5%. This is due to the presence of the parasitic component, which produces an additional resonance in the high-frequency band. This band is in close proximity to the resonance band generated by the radiating patch and then, the overall BW of the antenna is increased. Besides, when the parasitic element is added, the electromagnetic field of the MIMO system is redistributed, and the mutual coupling is, therefore, strongly affected. In this case, the coupling is significantly reduced, and the isolation across the operating band is lower than 25 dB, with the lowest value of 28 dB at 5.4 GHz.

### 3.3. MIMO Antenna with Parasitic Elements and Radiating Patches in Different Layers

To further improve the isolation, the parasitic elements are located in different layers of the radiating patch. The antenna configuration and optimized dimensions are given in Figure 1 and named Design–3.

**Figure 4:**
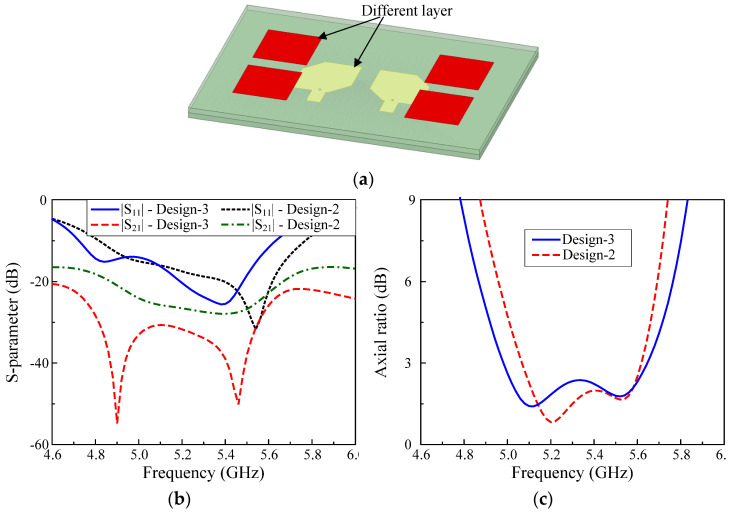
Simulated performance of the MIMO CP antennas with radiating patches and parasitic elements in the same layer (Design–2) and different layers (Design–3): (**a**) 3D–view, (**b**) S–parameter; (**c**) Axial ratio.

Figure 4 shows the performances of Design–2 and –3. As seen, the wideband performance is also achieved for Design–3, and the operating BW of this design is 12.4%, ranging from 4.98 to 5.64 GHz. However, the mutual coupling observes a significant reduction for Design–3. Here, the isolation across the operating BW is higher than 25 dB, while that for Design–2 is better than 20 dB. Besides, Design–3 also obtains an operating BW of 11% (4.98–5.56 GHz) in which the isolation is better than 30 dB.

**Figure 5:**
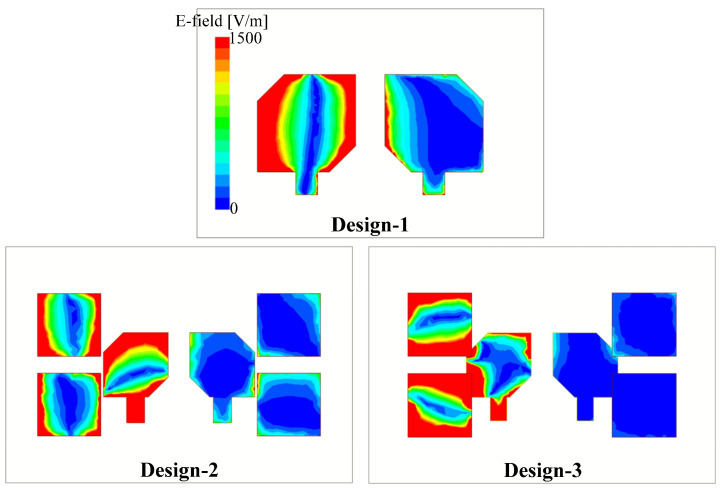
Simulated E-field distributions at 5.4 GHz for Design–1, –2, and –3.

To conclude, it can be seen that when the parasitic elements are located in different layers with the radiating patches, the mutual coupling between the MIMO elements is considerably decreased. This can be explained by observing the electric field distribution on Design–1, –2, and –3, shown in Figure 5. Design–1 exhibits a strong coupling from the excited patch to the non–excited one. It means that the coupling between the MIMO elements is very strong, leading to a low isolation value. With the presence of parasitic elements, the electromagnetic field is redistributed and for Design–2 and –3, more fields are coupled to the parasitic elements rather than the non-excited patch. Based on Figure 5, we can quantitatively realize that the best decoupling scenario is obtained for Design–3. It explains why this design has higher isolation than the others.

## 4. Antenna Discussion

The antenna design procedure has been thoroughly investigated in Section 3. In this Section, the key parameters, which strongly affect the antenna’s performance, are discussed. It is noted that when a parameter is studied, the other parameters are fixed at the optimized values.

### 4.1. Number of Parasitic Elements

**Figure 6:**
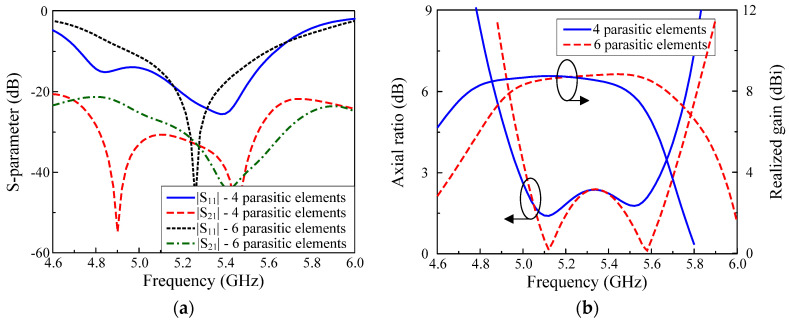
Simulated performance comparison of the proposed CP MIMO antenna with four and six parasitic elements: (**a**) S–parameter; (**b**) Axial ratio and gain.

Figure 6 shows the simulated results of MIMO antennas with four and six parasitic elements. In both cases, the operating BWs and the isolations within this band are almost similar. However, the antenna with four parasitic elements can achieve the best isolation of 50 dB, while that for the other is just 36 dB. In terms of gain, the six-parasitic-element antenna has a higher gain. It could be attributed to the larger antenna’s radiating aperture when more parasitic elements are utilized. This leads to the higher antenna’s aperture efficiency and the gain is, therefore, higher [24]. After considering the antenna’s performance and the antenna’s size, the design with four parasitic elements is chosen as the optimal design.

### 4.2. Matching Stub

**Figure 7:**
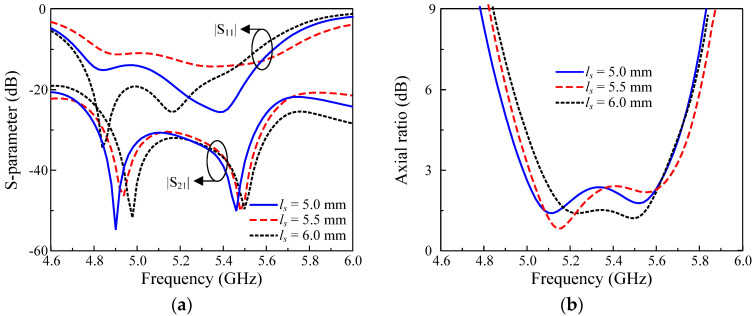
Simulated performance of the proposed CP MIMO antenna with different lengths of the stub (*l_s_*): (**a**) S–parameter; (**b**) Axial ratio.

To better control the impedance matching of the proposed design, additional stubs are utilized. The effect of the matching stub on the antenna is investigated and the simulated results are depicted in Figure 7. The data indicates that the length of the stub (*l_s_*) only has a significant impact on the |S_11_| parameter. On the other hand, the |S_21_| and AR are almost stable with the variation of *l_s_*. A similar phenomenon can be observed when changing the width of the stub (*w_s_*) and the simulated results are not presented for brevity.

### 4.3. CP Realization

**Figure 8:**
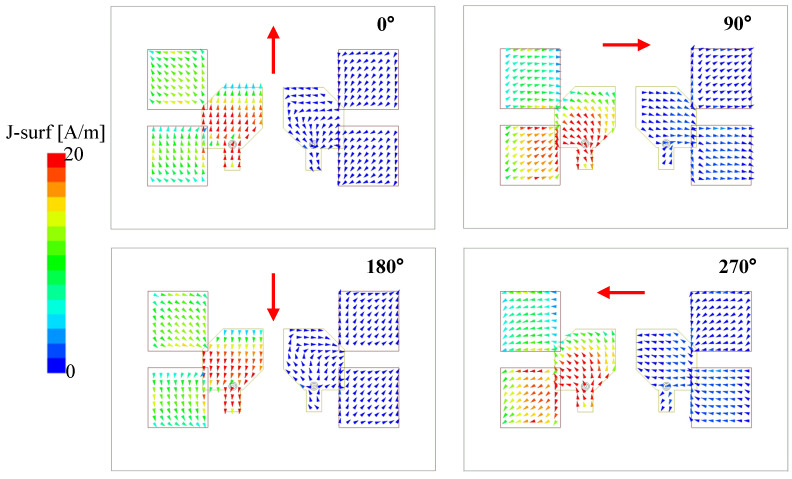
Simulated current distribution of the proposed CP MIMO antenna at 5.1 GHz.

To understand the CP realization, Figure 8 shows the current distribution on the antenna for different phases at 5.1 GHz. In this case, Port–1 is excited and the MIMO element on the left–side radiate. The current distribution indicates that when the phase changes from 0° to 270°, the vector current rotates in the clockwise direction. Thus, the antenna radiates LHCP waves. When Port–2 is excited, the antenna produces RHCP waves as the patches are truncated in a different set of corners. The current distributions for both cases are similar, but the directions of vector currents are opposite. Thus, the simulated results for Port–2 excitation are not presented.

## 5. Measured Results

**Figure 9:**
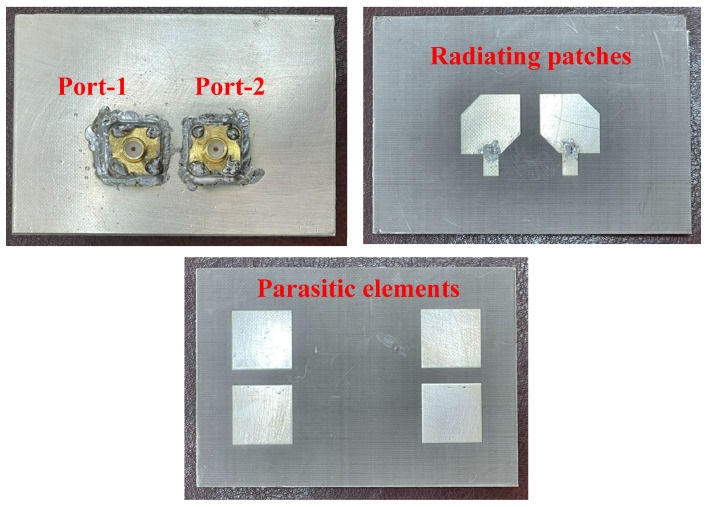
Photographs of the fabricated MIMO CP antenna with bottom layer (ground), middle layer (radiating patches), and top layer (parasitic elements).

An antenna prototype is fabricated and tested to confirm the design concept. Photos of the fabricated prototype are illustrated in Figure 9. We use the PNA Network Analyzer N5224A for S-parameter measurements, and the far-field features are conducted in an anechoic chamber. Overall, a similarity between the simulation and measurement is observed, which proves the design concept. In fact, the differences might come from the fabrication tolerances and the imperfection in the measurement setup.

### 5.1. S–Parameter and Far–Field Results

**Figure 10:**
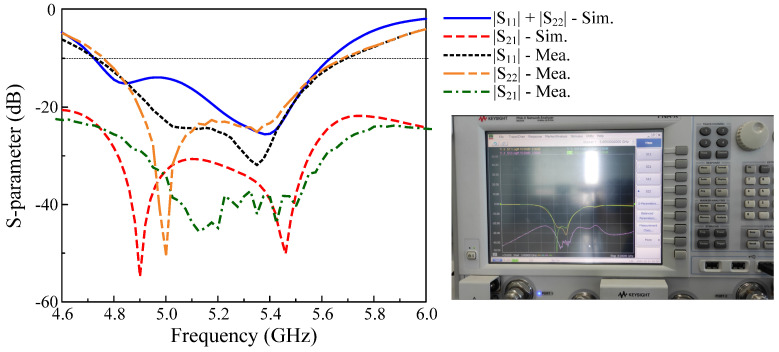
Simulated and measured S–parameter of the proposed MIMO CP antenna.

Figure 10 presents the S–parameter in terms of impedance matching and the isolation of the proposed MIMO antenna. As observed, the antenna exhibits wideband characteristics with overlapped −10 dB |S_11_| BW for Port–1 and –2 excitations of 17.3%, from 4.75 to 5.65 GHz. Across this band, the mutual coupling between the MIMO elements is significantly suppressed, and thus, the isolation is always better than 23 dB. The highest isolation is 45 dB at 4.95 GHz.

**Figure 11:**
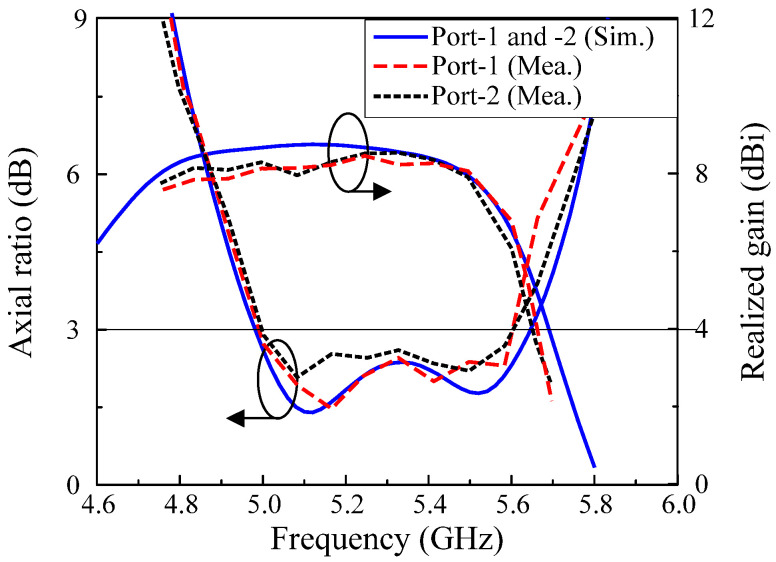
Simulated and measured ARs and realized gains of the proposed MIMO CP antenna.

Figure 11 depicts the AR and realized gain of the proposed design in the broadside direction. For far–field measurements, when one port is excited, the other is terminated with a 50–Ω load. It observes the overlapped 3–dB AR BW of 11.3%, from 5.0 to 5.6 GHz. This AR band is fully covered by the impedance–matching BW. The isolation within the AR band is better than 32 dB. Besides, the measured gain ranges from 6.0 to 8.5 dBic. The peak broadside gain is 8.5 dBic at 5.3 GHz.

**Figure 12:**
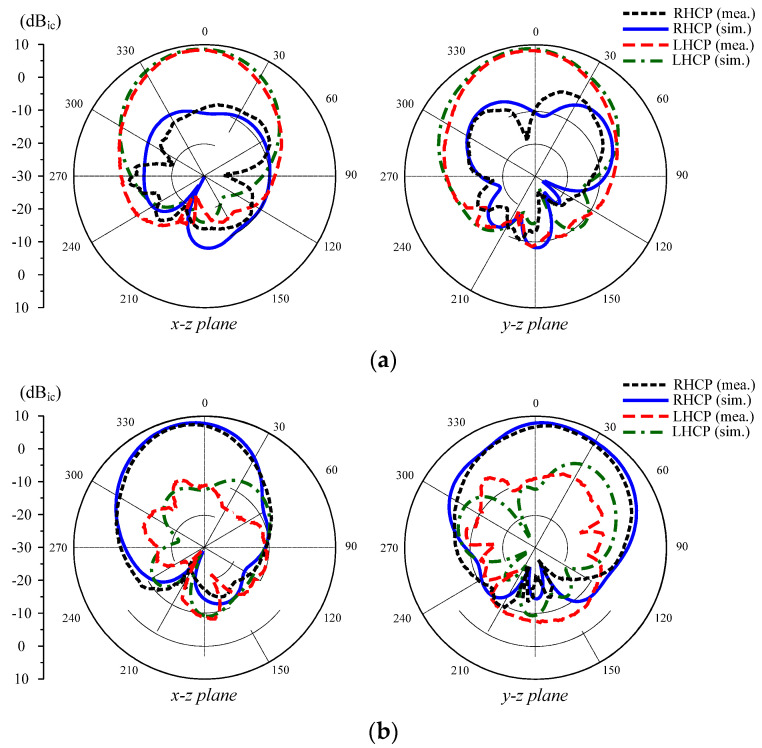
Simulated and measured radiation patterns of the proposed MIMO CP antenna. (**a**) 5.2 GHz and Port–1 excitation, (**b**) 5.5 GHz and Port–2 excitation.

The gain radiation patterns at 5.2 GHz for Port–1 and 5.5 GHz for Port–2 excitations are plotted in Figure 12. The data indicate that dual sense CP is realized based on the excitation port. In the broadside direction, the polarization isolation, which is the difference between the RHCP and LHCP gain levels, is higher than 16 dB. In addition, the front–to–back ratio defined by the gain difference in the front side and the back side is greater than 15 dB.

### 5.2. MIMO Parameters

In MIMO systems envelope correlation coefficient (ECC) is a crucial matrix to evaluate performance. The $ECC_{ij}$ tells how many antenna elements in the MIMO system are independent in their performance and can be calculated using S-parameters and far-field results by solving Equations (1) and (2), respectively [25,26,27,28,29,30].

(1) $$ECC_{ij}=\frac{{\left|R_{ii}^{*}*T_{ij}+T_{ji}^{*}*\;S_{jj}\right|}^{2}}{\left(1-{\left|R_{ii}\right|}^{2}-{\left|T_{ji}\right|}^{2}\right)\left(1-{\left|R_{jj}\right|}^{2}-{\left|T_{ij}\right|}^{2}\right)}$$

(2) $$ECC_{ij}=\frac{{\left|\iint_{0}^{4\pi }\left[\vec{R_{i}}\left(\theta ,\;\varphi \right)\times \vec{R_{j}}\left(\theta ,\;\varphi \right)\;\right]d\Omega \right|}^{2}}{\iint_{0}^{4\pi }{\left|\vec{R_{i}}\left(\theta ,\;\varphi \right)\right|}^{2}d\Omega \iint_{0}^{4\pi }{\left|\vec{R_{j}}\left(\theta ,\;\varphi \right)\right|}^{2}d\Omega \;}$$

Here, *i* and *j* stand for the antenna’s port number. $R_{ii}$ and $R_{jj}$ are the reflection coefficients at different ports. Meanwhile, $T_{ij}$ and $T_{ji}$ are denoting the transmission coefficients between two ports of the MIMO system. In Equation (2), the Ω expresses the solid angle of the radiation patterns and $\vec{R_{i}}\left(\theta ,\;\varphi \right)$ and $\vec{R_{j}}\left(\theta ,\;\varphi \right)$ are the 3-D patterns of the MIMO elements.

**Figure 13:**
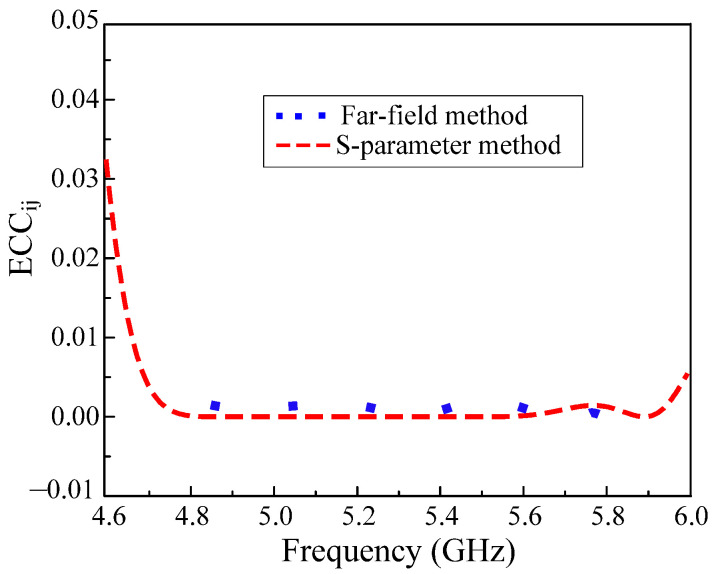
Envelope correlation coefficient of the proposed CP MIMO antenna.

For accurate results, the ECC is computed using both the S-parameter and the far-field methods, shown in Figure 13. The maximum $ECC_{ij}$ values for both calculation methods are noted to be 0.005 (close to the ideal value of zero) within the operating frequency. They are significantly lower than the acceptable value of 0.5. This verifies that the proposed MIMO antenna provides uninterrupted radiation patterns due to the effective usage of the parasitic elements for seamless connectivity.

**Figure 14:**
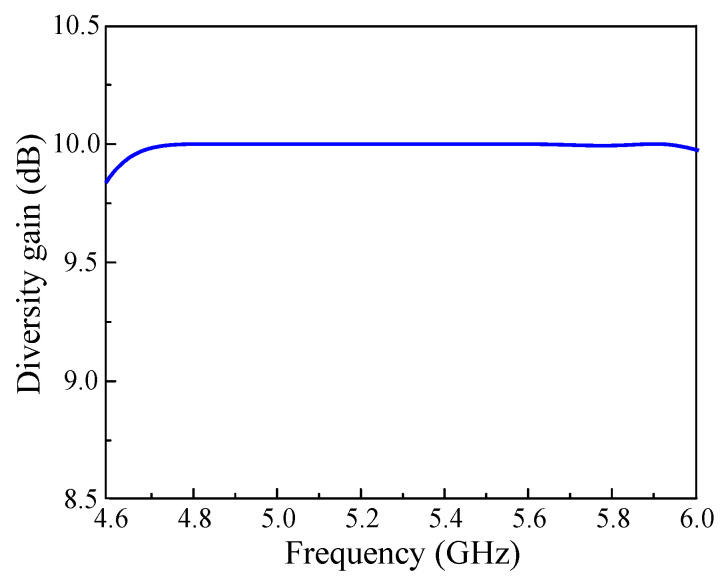
Diversity gain of the proposed CP MIMO antenna.

The diversity gain (*D_gain_*) of the proposed parasitic decoupler-based antenna is numerically computed using Equation (3) and shown in Figure 14. In fact, *D_gain_* explains the losses in power during transmission in the MIMO system. The *D_gain_* values are near to their identical value of 10 dB within the common bandwidth, which guarantees excellent diversity performance.

(3) $$D_{gain}=10\;\sqrt{1-|ECC_{ij}{|}^{2}}$$

Another critical parameter in the MIMO channel is its channel capacity loss ($CC_{loss}$), which can be estimated using the relation given in Equation (4).

(4) $$CC_{loss}{}_{\;}=-Log_{2}\det\left(m_{c}\right)$$

Here, $m_{c}$ is the correlation matrix which can be solved by:

(4a) $$m_{c}=\left[\begin{array}{cc} \sigma_{11} & \sigma_{12}\\ \sigma_{21} & \sigma_{22} \end{array}\right]$$

(4b) $$\sigma_{ii}=1-\left({\left|R_{ii}\right|}^{2}-{\left|T_{ij}\right|}^{2}\right)$$

(4c) $$\sigma_{ij}=1-\left(R_{ii}{}^{*}T_{ij}+T_{ji}R_{jj*}R_{jj}{}^{*}\right)$$

**Figure 15:**
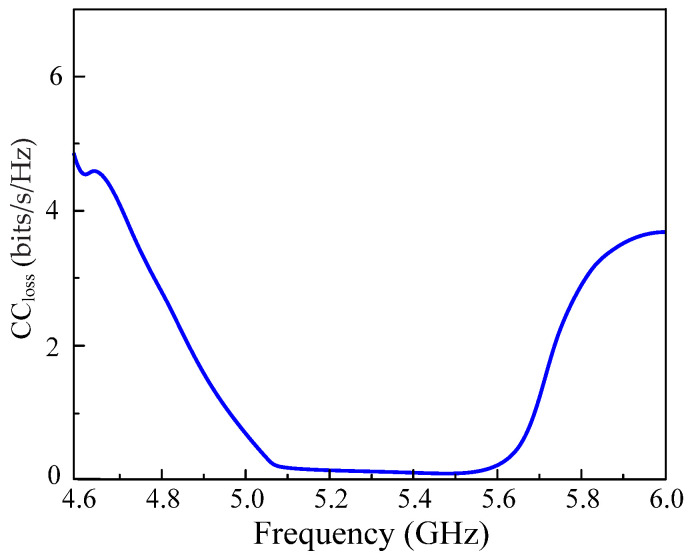
Channel capacity loss of the proposed CP MIMO antenna.

The $CC_{loss}$ of the proposed CP MIMO antenna is shown in Figure 15, which is very low at 0.15 bits/s/Hz in the desired frequency range of C–band and suitable for uninterrupted wireless communications with high throughput.

Besides ECC, DG, and CLL, other important parameters to evaluate the MIMO performance are the Mean Effective Gain (MEG) and Total Active Reflection Coefficient (TARC). These parameters are calculated based on the S-parameter [31]. In fact, when the reflection coefficient and transmission coefficient are, respectively, lower than −10 dB and −20 dB, good MEG and TARC values will be obtained. These conditions are satisfied by the proposed design and thus, MEG and TARC are not shown for brevity.

## 6. Conclusions

In this paper, a two–element CP MIMO using a microstrip patch and parasitic elements has been investigated. It has been shown that the parasitic elements have a significant influence on the antenna performance with respect to input impedance, and AR, as well as isolation. The measurements carried out on the fabricated prototype demonstrate that the proposed MIMO antenna has a broadband operation of 11.3% (5.3–5.6 GHz). Within this band, the isolation is always better than 32 dB with the best value of 45 dB, and the realized gain is from 6.0 to 8.5 dBi. Besides, the MIMO parameters in terms of the envelope correlation coefficient, diversity gain, and channel capacity loss are also investigated. The results prove the potential of the proposed design when used in MIMO C–band communication systems.

## Figures and Tables

**Table 1:** Performance comparison among MIMO CP antennas with unidirectional beam.

Ref.	Antenna Type	Size (λ_o_)	Spacing (λ_o_)	BW (%)	Isolation (dB)	Gain (dBi)
[4]	DRA	1.12 × 0.69 × 0.03	0.33	7.1	≥18	4.1
[5]	DRA	1.05 × 0.55 × 0.24	0.30	<5.0	≥26	5.0
[6]	Patch + FSS	1.58 × 1.58 × 0.70	0.67	18.5	≥23	14.1
[8]	Patch	1.25 × 0.83 × 0.01	0.06	1.9	≥20	6.1
[9]	Patch	1.77 × 0.51 × 0.03	0.25	1.0	≥26	5.3
[12]	Patch + MS	0.95 × 0.54 × 0.05	0.18	13.7	≥20	5.8
[13]	Patch + MS	1.83 × 1.83 × 0.05	0.36	16.8	≥30	11.0
[14]	Patch + Parasitic element	0.95 × 0.71 × 0.05	0.09	8.3	≥26	6.2
Prop.	Patch + Parasitic element	1.41 × 0.97 × 0.05	0.08	11.3	≥32	8.5

**Table 2:** Optimized dimensions of the Design–1, –2, and –3 (unit: mm).

Parameters	Design–1	Design–2	Design–3
*w*_1_	17.0	15.0	15.0
*l_c_*	4.6	4.6	5.0
*l_f_*	6.6	6.6	6.6
*l_s_*	4.0	6.0	5.5
*w_s_*	3.8	2.8	3.8
*d*_1_	5.0	5.0	5.0
*d*_2_		36.2	32.4
*w*_2_		14.6	14.8
*s*		4.0	4.0

## Data Availability

Not applicable.
